# IL-35: A Novel Immunomodulator in Hepatitis B Virus-Related Liver Diseases

**DOI:** 10.3389/fcell.2021.614847

**Published:** 2021-03-11

**Authors:** Xuefen Li, Xia Liu, Weilin Wang

**Affiliations:** ^1^Key Laboratory of Clinical In Vitro Diagnostic Techniques of Zhejiang Province, Department of Laboratory Medicine, The First Affiliated Hospital, Zhejiang University School of Medicine, Hangzhou, China; ^2^Hangzhou Global Scientific and Technological Innovation Center, Zhejiang University, Hangzhou, China; ^3^Key Laboratory of Precision Diagnosis and Treatment for Hepatobiliary and Pancreatic Tumor of Zhejiang Province, Division of Hepatobiliary and Pancreatic Surgery, Department of Surgery, Clinical Research Center of Hepatobiliary and Pancreatic Diseases of Zhejiang Province, The Second Affiliated Hospital, Zhejiang University School of Medicine, Hangzhou, China

**Keywords:** chronic hepatitis B, interleukin-35, cytotoxic T lymphocytes, induced regulatory T-cells, immunopathology

## Abstract

Chronic hepatitis B virus (HBV) infection is a risk factor for liver cirrhosis (LC) and hepatocellular carcinoma (HCC), however, little is known about the mechanisms involved in the progression of HBV-related diseases. It has been well acknowledged that host immune response was closely related to the clinical outcomes of patients with HBV infection. As the factors closely related to the immunomodulatory process, cytokines are crucial in the cell-cell communication and the host responses to HBV infection. Recently, a newly discovered cytokine, designated as interleukin-35 (IL-35), has been proved to be essential for the progression of chronic HBV infection, the development of cirrhosis, the transformation of cirrhosis to HCC, and the metastasis of HCC. Specifically, it showed various biological activities such as inhibiting the HBV-specific cytotoxic T lymphocyte (CTL) proliferation and cytotoxicity, deactivating the immature effector T-cells (Teffs), as well as delaying the proliferation of dendritic cells. It regulated the immune responses by acting as a “brake” on the activation of Teffs, which subsequently played important roles in the pathogenesis of various inflammatory diseases and malignancies. In this review, we focused on the most recent data on the relationship between IL-35 and chronic HBV infection, LC and HCC.

## Introduction

Hepatitis B virus (HBV) infection is still a threat to health worldwide causing chronic infection among 250 million individuals (Lee and Banini, 2019). Patients with HBV infection may develop hepatic failure, liver cirrhosis (LC), and primary hepatocellular carcinoma (HCC), resulting in a death toll of more than 1 million annually (Polaris Observatory Collaborators, 2018; Lee and Banini, 2019). The pathogenesis of HBV infection is rather complicated, which is influenced by several factors including HBV genotype, viral variation and replication. In addition, host factors (e.g., sex, age, and immune status) and other exogenous factors (e.g., hepatitis viral co-infection and alcoholism) were also reported to be involved (Tong et al., 2010; Tang et al., 2018; Terrault et al., 2018; Vlachogiannakos and Papatheodoridis, 2018). Therefore, various manifestations of HBV infection can be seen clinically at different stages (Table 1). HBV is infected through person-to-person transmission at birth or after birth. Although the vaccines against HBV have been developed in the 1980s, about 1/3 of the population worldwide still showed a previous or existing HBV infection in serological tests (World Health Organization, 2013). Globally, viral hepatitis caused 1.34 million deaths in 2015. This result is similar to tuberculosis deaths (1.37 million) and is higher than HIV infections (1.06 million) and malaria (0.44 million). About 96% of these people died from complications of chronic hepatitis and most of them (66%) were diagnosed with chronic hepatitis caused by HBV. It was recognized as the disease with the highest burden by the WHO Western Pacific region (which includes 37 countries) in 2016 (World Health Organization, 2017; Seto et al., 2018).

**TABLE 1:** Clinical features of patients infected with HBV at different stages (Tong et al., 2010; Tang et al., 2018; Terrault et al., 2018; Vlachogiannakos and Papatheodoridis, 2018).

Clinical parameters	Stages of HBV infection					
	Occult HBV infection	Inactive carrier	Acute self-limit HBV infection	Active chronic HBV infection	HBV-related LC	HBV-related HCC
Virological markers	HBsAg (−)	HBsAg (+)	HBsAg (+)	HBsAg (+)	HBsAg (+)	HBsAg (+)
	HBeAg (−)	HBeAg (−)	HBeAg (+)	HBeAg (±)	HBeAg (±)	HBeAg (±)
	Anti-HBc (+)	Anti-HBc (±)	Anti-HBc (±)	Anti-HBc (±)	Anti-HBc (±)	Anti-HBc (±)
	Anti-HBe (±)	Anti-HBe (±)	Anti-HBe (±)	Anti-HBe (±)	Anti-HBe (±)	Anti-HBe (±)
	HBV-DNA (+)	HBV-DNA (<2,000 IU/ml)	HBV-DNA (+)	HBV-DNA (+)	HBV-DNA (±)	HBV-DNA (±)
Liver biochemistry	Normalization	Normalization	↑	↑↑	Normalization/↑	Normalization/↑/↑↑
Liver histopathology	No liver damage	Low grade inflammation	Minimal or temple liver damage	Increased liver damage	Increased liver damage	Increased liver damage
Antiviral therapy	No	No	No	Yes	Yes	Yes

*HBV, Hepatitis B virus; HBsAg, Hepatitis B surface antigen; HBeAg, Hepatitis B e antigen; Anti-HBc, Antibody to HBcAg; Anti-HBe, Antibody to HBeAg; (−): Negtive; (+): Positive; (±): Positive or Negative; ↑: Increased; ↑↑: Significantly increased.*

To date, the pathogenesis of HBV infection is still not well defined. As is known to all, persistent HBV infection has been acknowledged to be closely associated with inadequate immune responses. Both innate and adaptive immunity were reported to be involved in anti-HBV immune response. During early stage of virus infection, innate sensing of viruses can occur through Toll-like receptors (TLRs) and cytosolic sensors that recognize viral DNA and RNA, then transmit a warning message to initiate downstream signals and activate effector components. In the later phase, antigen-presenting cells (APCs), including macrophages and dendritic cells (DCs), initialize the virus-specific adaptive immunity characterized by activation of T helper (Th) lymphocytes and secretion of various cytokines, which then mobilize the CD8^+^ cytotoxic T lymphocytes (CTLs) to kill the HBV-infected hepatocytes (Bertoletti and Gehring, 2006). Previous work showed that cell-mediated immunity is critical for clearance of HBV infection, in particular, robust and multiple epitope-specific CD8^+^ T cell responses are necessary for the spontaneous resolution of HBV infection (Thimme et al., 2003). Moreover, HBV-specific CD4^+^ T cells are also important in determining the outcome of acute HBV infection, as shown in a chimpanzee study that the depletion of CD4^+^ T cells abrogated the function of CD8^+^ T cells and resulted in chronic HBV infection (Asabe et al., 2009). In addition to T-cell responses, neutralizing antibodies produced by B cells are believed to play an integral role in of HBV detection and resolution. Unlike the adaptive immune system, the innate lymphoid cells (ILCs) mainly localized at mucosal surfaces acting as sentinel cells, where release of their cytokine suite inhibits establishment and spread of HBV virus infection (Yang et al., 2015). Importantly, there is growing evidence that cytokine-mediated immune responses play an important role in determining clinical outcomes during HBV viral control and virus-induced hepatic injury (Dandri and Locarnini, 2012; Seetharam et al., 2014; Li et al., 2016).

Interleukin (IL)-35 is a newly identified cytokine of the IL-12 family and a potent immunosuppressive cytokine secreted by regulatory T (Treg) cells and the regulatory B (Breg) cells (Xiang and Xie, 2015). IL-35 and iTr35 cells develop a positive feedback loop to interact with each other, as iTr35 cells generation can be induced by IL-35, while more IL-35 is further secreted by iTr35 cells (Collison et al., 2010). Like transforming growth factor-β (TGF-β) and IL-10, IL-35 can also induce the development of induced regulatory T cell (iTreg) population (Collison et al., 2007). Moreover, IL-35 stimulation increased the inhibitory function of CD4^+^CD25^+^CD127^dim/–^ Tregs by reducing cellular proliferation and enhancing IL-35/IL-10 productions (Mitani et al., 2015; Shi et al., 2015). Thus, the current researches suggested that the elevated proportion of Tregs might be the major source of IL-35 enhancement in the serum of patients with CHB. Besides, IL-35 also induces Breg cells formation *in vivo* and promotes their conversion to a unique Breg subset that produces IL-35 (IL-35^+^ Breg) (Wang et al., 2014), and inhibit the proliferation and function of effector T-cells (Teffs) (Collison et al., 2007; Collison et al., 2010). A novel immunosuppresive subset named CD8^+^ Tregs were reported in both human and mice, exerting immunoregulatory function through secreting cytokines (Flippe et al., 2019). IL-35 has also been described as expressed and playing a suppressive role for tumor-associated CD8^+^ Tregs (Olson et al., 2012; Zhang et al., 2019). Although very few papers were published on CD8^+^ Tregs function in HBV infection, based on the anti-inflammation role of CD8^+^ Tregs in human disease (Vuddamalay and van Meerwijk, 2017), we hypothesize that IL-35 secreted by CD8^+^ Tregs in CHB patients may similarly contributed to immune tolerance and exhaustion state of CTLs, therefore, targeting IL-35 secreted CD8^+^ Tregs may provide peculiar thought for treatment of HBV infection. In conclusion, IL-35 can induce the generation of iTr35, Bregs and possibly CD8^+^ Tregs, which function as an immunosuppressive factor in immune mediated progression of chronic hepatitis B.

The effect of IL-35 on pro-inflammatory cytokines were also reported to be involved in the pathogenesis of HBV. For instance, IL-35 stimulation notably reduced concentrations of interferon-gamma (IFN-γ), IL-1β, IL-6, and IL-8 produced by PBMCs, indicating IL-35 inhibited cytokines-induced antiviral immunity to HBV. Furthermore, the reduction of pro-inflammatory cytokine secretion in HBsAg and IL-35 co-stimulated PBMCs was accompanied by the decreased phosphorylation of STAT1 in comparison of HBsAg stimulation only group (Shao et al., 2017). Previous studies demonstrated that IL-35 showed inhibitory effects on the HBV-specific CTL response, which affected HBV clearance and modulated the pathogenesis of HCC (Li et al., 2015; Xiang and Xie, 2015; Xue et al., 2019). To our best knowledge, although more researches are exploring the role of IL-35 in human disease, few studies have been focused on the roles of IL-35 in HBV infection. In this review, we summarized the possible immunoregulatory roles of IL-35 in the pathogenesis of CHB, LC and the hepatocarcinogenesis related with HBV infection. It contributed to the proposal of potential strategies for reversing exhaustion of T cells and the treatment programs of CHB.

## Biological Properties of IL-35

**FIGURE 1:**
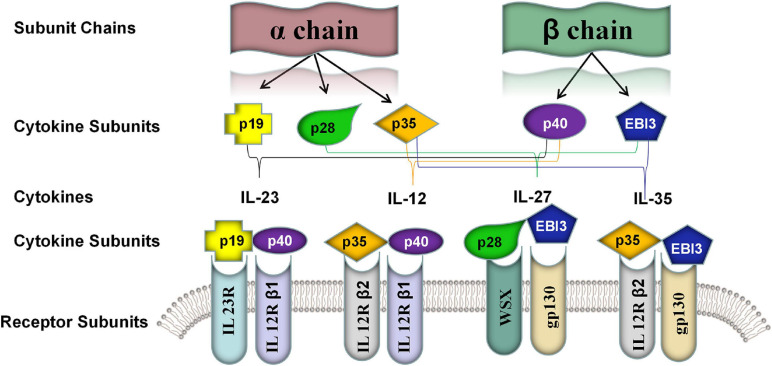
Five known members of the IL-12 family cytokine subunits and their corresponding receptor subunits. The IL-12 family was characterized by different assembling of a α chain consisted of p19, p28, and p35 and β chain consisted of p40 and EBI3. The members of IL-12 shared the subunits and their cognate receptors including IL-23R, IL-12R-β1, IL-12R-β2, IL-27R [WSX] and gp130. IL-23: p19 and p40; IL-12: p35 and p40; IL-27: p28 and EBI3; IL-35: p35 and EBI3; IL-39: p19 and EBI3.

IL-35 is a novel cytokine of the IL-12 family (Xiang and Xie, 2015). Each of IL-12 family members (e.g., IL-12, IL-23, IL-27, and IL-39) is a heterodimeric cytokine composed of α chain (i.e., p19, p28, and p35) and β chain including p40 and Epstein-Barr virus-induced gene 3 [EBI3], which contain common subunits and receptors (Figure 1; Bastian et al., 2019). Structurally, it was composed of the IL-27β chain EBI3 subunit and the IL-12α chain p35 subunit. Although IL-12 family members share similar subunit structures, they have significantly different biological properties. Unlike other members of the IL-12 family, IL-35 showed strong immunosuppressive features (Collison et al., 2007; Su et al., 2018).

Human IL-35, expressed in response to inflammatory stimuli, was reported to inhibit proliferation and function of Teffs and convert the conventional CD4^+^ T cells (Tconv) to highly inhibitory IL-35 induced regulatory T cell (iTr35), which further induced immune tolerance in hosts, and promoted viral infection (Collison et al., 2009; Shen et al., 2014). In a previous study, Collison et al. (2007) demonstrated that IL-35 was implicated in the conversion of naive T cells into iTr35 in human or mouse. Transcriptional analysis revealed that there were highly restricted genes characteristics of iTr35 (CD4^+^Foxp3^–^EBI3^+^p35^+^IL10^–^TGFβ^–^), which were distinct from the other two types of Tregs (iTr), TGF-β-iTr (CD4^+^Foxp3^+^TGF-β^+^) and IL-10-iTr (CD4^+^Foxp3^+^IL-10^+^ or CD4^+^Foxp3^negative^IL-10^+^), which were regulated by differential transcriptional regulators, respectively (Brockmann et al., 2018). Gene expression analysis indicated that IL-35 was distributed in broad tissues, and was a responsive anti-inflammatory cytokine induced by pro-inflammatory cytokines in a variety of non-T cells such as tumor cells, Bregs, dendritic cells (DCs), endothelial cells, smooth muscle cells, and monocytes (Li et al., 2012; Choi et al., 2015; Dixon et al., 2015).

**FIGURE 2:**
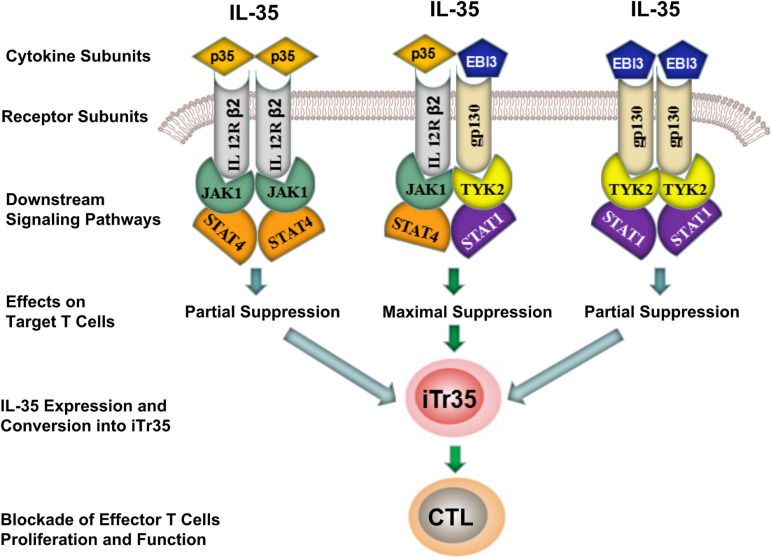
Differences of inhibitory effects between homodimeric and heterodimeric IL-35. IL-35 signal inhibited the differentiation of effector T-cells (Teffs) by activating the intracellular JAK-STAT signaling pathway. Although both homodimeric (EBI3/EBI3 and p35/p35) and heterodimeric (EBI3/p35) IL-35 were capable of producing iTr35, the later showed a maximal suppressive activity by producing iTr35 and blocked the proliferation and function of Teffs. CTL, Cytotoxic T lymphocyte; iTr35, IL-35-producing Treg cells; JAK, Janus kinase; STAT, Signal transducer and activator of transcription.

The transmission of IL-35 signals is mainly relied on a heterodimer formed by gp130 and IL-12Rβ2 receptor chain subunits, or a homodimer of IL-12Rβ2 or gp130 subunits. IL-35 did not fully induce iTr35 transformation despite the fact that homodimeric IL-35 (i.e., EBI3/EBI3 and p35/p35) could block Teffs differentiation by activating one of its signaling pathways. In contrast, heterodimeric IL-35 (EBI3/p35) was capable of inducing iTr35 (Figure 2). Such difference was mainly associated with the downstream receptor signaling (Huang et al., 2017). The maximal suppressive activity of IL-35 involves the formation of a unique heterodimers of IL12Rβ2 and gp130, which subsequently binds the downstream transcriptional factors (i.e., STAT1 and STAT4). These combined transcriptional factors could uniquely bind with different sites in the *p35* and *EBI3* gene promoters. Thus, heterodimeric IL-35 contributed to the expression of p35 and EBI3, which formed a positive feedback loop modulating the secretion of IL-35 (Collison et al., 2012). As previously described, IL-35 rather than IL-12 or IFN-γ promoted the conversion of Tconv cells into iTr35. Besides, it could induce STAT1 and STAT4 activation, which demonstrated that IL-35 was highly specific to STAT1 and STAT4 (Thierfelder et al., 1996; Hoey et al., 2003; Collison et al., 2012; Louis, 2016). In future, more studies focusing on the composition and function of these downstream receptors in the IL-35 signaling pathways are required to further illustrate the potential biological properties of IL-35.

Different from IL-12 family cytokines, the inhibitory activity of IL-35 was primarily on the CTL/Th cell proliferation and effector function. Among the subpopulations of Th cells, both Th1 and Th2 have been implicated in the pathogenesis of hepatic inflammation during HBV infection (Bertoletti and Ferrari, 2012). On one hand, IL-35 significantly inhibited immune activation of Th1/CTL cells together with secretion of IL-2 and IFN-γ in inflammation and related diseases of animal disease models (Guo et al., 2017). On the other hand, IL-35 showed attenuating effects on allergen-specific CD4^+^ memory/effector Th2 cell-mediated airway inflammation (Huang et al., 2011). Th2 cells are in favor of anti-inflammation partly depending on the production of IL-10, as well as the secretion of other cytokines (e.g., IL-4, IL-5, and IL-13), which were induced by IL-35 administration (Guo et al., 2017; Teymouri and Pirro, 2018). IL-10 is recognized as a key cytokine regulating the immune response to HBV infection. Das et al. (2012) reported that the down-regulation of IL-10 restores the function of exhausted HBV-specific CD8^+^ T cells (Das et al., 2012; Liu et al., 2016). Moreover, IL-10 might substantially affect the antiviral immune response, as it inhibits the production of pro-inflammatory cytokines such as IFN-γ, tumor necrosis factor-alpha (TNF-α), IL-1β, and IL-6 (Rybicka et al., 2020). As recent studies suggest that rIL-35 fusion proteins can induce Breg cells to secret IL-10 (Wang et al., 2014), hence, is possible that IL-35 induce Th2 and Breg cells to secret IL-10, which was involved in immunologic tolerance in CHB patients.

Furthermore, IL-35 stimulation was shown to inhibit the differentiation of HBV core-specific (CD4^+^CD25^+^Foxp3^+^) Tregs into a IL-17-secreting CD4^+^ T cells (Th17), and both of the Th subsets have been shown to be associated with disease progression or liver damage in CHB patients (Zhang et al., 2011; Cheng et al., 2015; Teng et al., 2019; Yang et al., 2019). Study indicated that the decreased Tregs/Th17 ratio and increased TGF-β1/IL-17 ratio can be used independently to predict the prognosis and disease progression and may be associated with the survival and disease progression of patients (Yu et al., 2014). Besides, *in vitro* IL-35 stimulation reduced Th17 cell subset and Th17 cytokine production in CD4^+^ T cells from acute hepatitis B (AHB) patients (Teng et al., 2019). Moreover, *in vivo* treatment of IL-35 decreased the HBV-induced liver injury and reduced hepatic NK cells and HBV peptides-induced Th17 cells in HBV plasmid injected mouse model (Teng et al., 2019). Taken together, IL-35 was crucial for regulation of peripheral and hepatic HBV peptides-induced Th17 cells *in vitro* and *in vivo*, which might subsequently modulated hepatocytes damage and liver inflammation during acute HBV infection.

In CHB patients, the major biological roles of IL-35 are mainly associated with the disruption of the balance between the immunocytes, which triggered the increase of immunosuppressive cells in number and functional disorder. Meanwhile, IL-35 could trigger the decline of effector cells and the functional attenuation. It finally induced the pathogenesis and even the progression of inflammation-related diseases.

## IL-35 and CHB

For patient with HBV infection, virus elimination or persistency was closely related to the appropriate immune responses. Cytokines are known as important chemical mediators regulating the differentiation, proliferation and function of immune cells, which could trigger the pathogenesis of liver disease and affect various clinical presentations (Sodsai et al., 2013; Li et al., 2016; Nitschke et al., 2016). Accumulating evidence indicated that serum IL-35 showed significant increase in patients with chronic HBV infection. According to the previous description, serum IL-35 was positively correlated with HBV DNA load and Tregs levels, and there was a negative correlation between IL-35 and circulating effector CD8^+^ T cells (Li et al., 2015; Moreno-Cubero and Larrubia, 2016; Nitschke et al., 2016). These supported a potential role of IL-35 and iTr35 cells in maintaining the immune tolerance during HBV infection. However, the exact role of IL-35 is still not completely understood, and further experiments and clinical trials are required to illustrate the role of IL-35 in this process.

**FIGURE 3:**
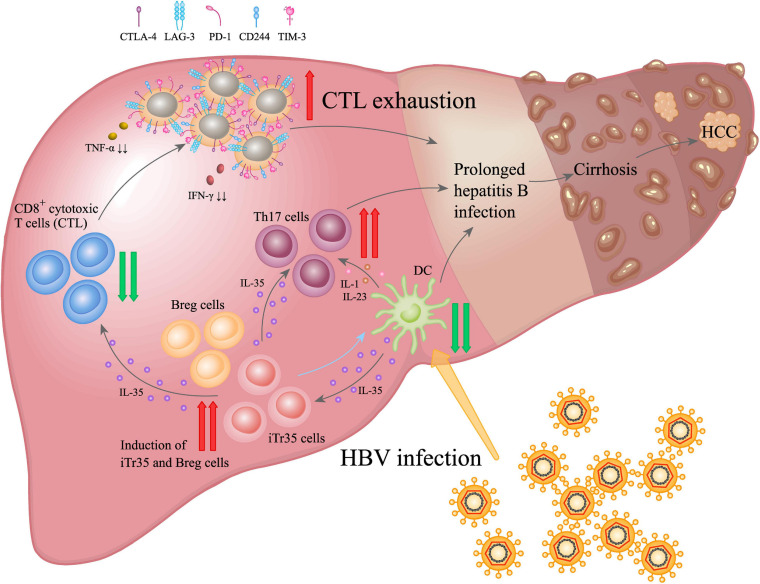
IL-35 promoted the dynamic interaction among disease progression of HBV infection driven by immunocytes. During chronic HBV infection, IL-35 up-regulation appeared as a primary effector in suppressing CTL-mediated immunity response and reducing dendritic cells (DCs) activation. It also induced fibrosis via activating Th17 and Tregs. IL-35-mediated regulatory function was closely related to the inhibition of Teffs proliferation and function, and modulation of T-cell differentiation, as well as induction of iTr35 and Breg (IL-35 and iTr35 develop a positive feedback loop to interact each other: iTr35 can be induced by IL-35, meanwhile, more IL-35 would be further secreted by iTr35 cells), which resulted in persistent HBV infection, liver cirrhosis and hepatocellular carcinoma (HCC).

As a responsive anti-inflammatory cytokine, IL-35 may have multifunctional roles involved in HBV-induced liver diseases (Figure 3). Elevation of IL-35 was reported to contribute to immunosuppression in CHB by modulating the balance between Th17 and Tregs (Collison et al., 2010; Yang et al., 2019). Some studies showed that although the plasma IL-35 levels and circulating HBV peptide-induced Th17 frequency showed significant increase in patients with hepatitis B, IL-35 expression was negatively correlated with liver inflammation (Teng et al., 2019; Yang et al., 2019). Besides, *in vivo* IL-35 administration down-regulated HBV peptides-induced Th17 cells in the liver, suggesting IL-35 might be a novel mediator associated with hepatocytes damage and liver inflammation by regulating HBV peptides-induced Th17 cells during acute HBV infection (Teng et al., 2019). Our recent study also indicated that only a small amount of IL-35 could trigger significant inhibition on the proliferation of CD4^+^CD25^–^CD45RA^+^ T cells. Similarly, it could significantly inhibit DCs proliferation and promote secretion of IL-10 and IL-6. Upon synergic stimuli by HBV core antigen peptide (Collison et al., 2007, 2010; Olson et al., 2012; Wang et al., 2014; Mitani et al., 2015; Shi et al., 2015; Shao et al., 2017; Vuddamalay and van Meerwijk, 2017; Flippe et al., 2019; Zhang et al., 2019) to the peripheral blood mononuclear cells (PBMCs) of CHB patients *in vitro*, IL-35 significantly inhibited the proliferation of HBV-specific CTL cells and IFN-γ secretion (Li et al., 2015).

The regulation of IL-35 on virus-specific Tregs/Th17 balance may lead to viral persistence in chronic HBV infection. Zhou et al. (2015) showed that the IL-35 level was significant higher in CD4^+^ T cells in patients with chronic HBV infection compared with that of the healthy individuals. Meanwhile, a high IL-35 level was related to a high HBV cccDNA replication in chronic HBV infection group, which indicated that HBV could induce the production of IL-35 by CD4^+^ T cells. Moreover, HBV infection also involved in the activation of immune system, while IL-35 showed immunosuppressive effects upon onset of chronic HBV infection. In a previous study, Tao et al. (2018) reported that IL-35 played a novel role in HBV replication. It had shown that IL-35 participated in stimulating the transcription and replication of HBV through interacting with hepatocyte nuclear factor 4α (HNF4α). Recombinant human IL-35 (rhIL-35) promoted HBV DNA replication and secretion of HBV core antigen, hepatitis B surface antigen (HBsAg), and hepatitis B e antigen (HBeAg). This implied that HNF4α may be a target for IL-35. On this basis, mutation of the *HNF4*α binding site on the HBV core promoter or silencing *HNF4*α may abolish the IL-35-induced enhancement on HBV replication.

Among the diseases caused by viral infections, high IL-35 was found to enhance the immunosuppressive activity of Tregs, and was positively correlated with the severity of diseases (e.g., CHB) (Damo and Joshi, 2019). According to previous description, Tregs and Th17 cells were closely interacted in the pathogenesis of viral infection. Specifically, Th17 cells contributed to the immune activation and disease progression, while Tregs may inhibit such process and play crucial roles in the maintenance of immune stability (Wan et al., 2020). In cases of chronic viral infection, there was high expression of Foxp3 and low expression of CD127 in the Tregs, which may directly promote the immune tolerance through direct cellular contact and inhibitory factors (e.g., IL-10 and IL-35) (Klein et al., 2010; Karkhah et al., 2018). In Th17 cells, there was high expression of transcriptional factor retinoic acid-related orphan receptor γt (RORγt). Besides, it could secret the IL-17 and IL-22, which induced the hepatic inflammation and fibrosis associated with the HBV infection (Zhang et al., 2011; Zúñiga et al., 2013; Nikoopour et al., 2015; Paquissi, 2017). Tregs and Th17 imbalance was reported to be associated with the liver injury, and was also a risk factor for the hepatic fibrosis and HCC pathogenesis in CHB patients (Li et al., 2017; Liu et al., 2017). Furthermore, effective anti-viral therapy could down-regulate the response of CD4^+^ T cells or CD4^+^CD25^+^CD127^dim/–^ Tregs to IL-35 stimulation *in vitro* (Yang et al., 2019). Studies on Tregs in HBV infection indicated that the circulating CD4^+^CD25^+^ Tregs population was expanded in the persistence HBV infection patients (Manigold and Racanelli, 2007). It has been shown that Tregs could inhibit the HBV-specific CTL proliferation and the secretion of cytokines, which plays a role in suppressing antiviral T cell responses and aiding viral persistence (Knolle et al., 2015; Jung and Shin, 2016). Interestingly, in hepatitis B patients received anti-viral therapy, there was a decline of Tregs in the peripheral blood, accompanied with recovery of Teffs response. This contributed to the prediction of the efficiency treatment regimens (Sprengers et al., 2007; Stoop et al., 2007). In a recent study, Tregs derived IL-35 promoted the expression of CD4^+^ Tconv, CD8^+^ T cells and B lymphocytes inhibitory receptor, such as programmed cell death protein 1 (PD-1), T cell immunoglobulin and mucin domain 3 (TIM-3), cytotoxic T Lymphocyte-associated antigen-4 (CTLA-4) and lymphocyte activation gene 3 (LAG-3), which played important roles in the tolerance of infection (Sullivan et al., 2020).

The studies discussed above indicated that IL-35 played a double-edged sword role in the pathogenesis of CHB. Specifically, it can induce the generation of Th17 cells, which thereby regulating the inflammation. Moreover, IL-35 played an important role in modulating the differentiation of Tregs with anti-inflammatory functions. IL-35 could trigger the up-regulation of Tregs and regulate the specific Tregs/Th17 balance, which may contribute to viral persistence in chronic HBV infection. More importantly, CD4^+^CD25^+^ Tregs are able to inhibit activation and proliferation of effective CD4^+^ or CD8^+^ T cells, which then suppresses the amplification of HBV-specific CTLs and secretion of cytokines. Therefore, the immunosuppressive effects of IL-35 demonstrate that IL-35 can lead to imbalance of immune response and CTL exhaustion, leading to a poor immune response to HBV. In general, the above results indicated a potential mechanism of IL-35-induced immunoregulation in chronic HBV infection.

## IL-35 and HBV-Related LC

Chronic HBV infection may induce deterioration of liver function through triggering liver injury and persistent liver inflammation, which subsequently resulted in paraplasm in the hepatic connective tissues and the consequential HBV-related liver fibrosis (HBV-LF), LC and even HCC (Xu et al., 2012). Various studies have suggested the pathology of HBV-related LC involves various immune components, especially immune cells and cytokines (e.g., IL-35) (Li et al., 2016; Tsai et al., 2018; Luo et al., 2019).

As mentioned above, IL-35 could directly inhibit the proliferation and function of Teffs, as well as the differentiation of Th17 cells. Meanwhile, it could expand the immunoregulatory reactions through the release of IL-35 from iTr35, which enhanced the tolerance to infection (Song and Ma, 2016). According to previous study, IL-35 pathway was closely associated with the progression of CHB to LC (liver cirrhosis), which implied that IL-35 may involve in the HBV-related LC (Shi et al., 2015). Additionally, the serum IL-35 in HBV-related LC patients was significantly higher compared with that of the normal control group, while serum IL-35 was positively correlated with IL-17, IL-22, and IL-33 which all acted as multifunctional roles involved in HBV infection (Wang et al., 2012; Zhao et al., 2014). Sun et al. (2012) indicated that the elevation of Th17 cells in hepatic cirrhosis patients would promote the activation of stellate cells in liver, which finally triggered the disease progression. At the early stage of HBV infection, Th17 could secret pro-inflammatory factors including IL-17, TGF-β and IL-22. It seems that IL-35 in LC may play a role in sustaining the pathologic process, since increase of IL-35 and other inflammatory cytokines (e.g., TGF-β, IL-22, IL-23, IL-31, and IL-33) can augment and extend hepatic inflammation (Cheng et al., 2015; Ming et al., 2015). For example, IL-35 can collaborate with TGF-β to exert immunoregulatory function and then present effective and maximal anti-inflammatory outcome (Slawek et al., 2020). Previous studies found that the mRNA and protein expression of TGF-β1 were significantly up-regulated in both the plasma and liver tissue in patients with fulminant liver failure (Miwa et al., 1997), and overexpression of TGF-β1 delayed liver regeneration and promoted perisinusoidal fibrosis and hepatocyte apoptosis in the rat model of fulminant liver failure (Yoshimoto et al., 2005). Mechanically, IL-35 could prevent the binding of TGF-β and its receptor, which inhibit phosphorylation of Smad3, a downstream effector of TGF-β receptor, thereby preventing the differentiation of Th17 cells and the synthesis of IL-17 in HBV-related LC patients (Ming et al., 2015).

All these indicated that IL-35 involved in the pathogenesis of the HBV related hepatic fibrosis. Therefore, attention should be paid to the side effects of IL-35 inhibition. Indeed, there is a long way for the application of such regimen for treating fibrotic diseases. On this basis, it is necessary to understand the relationship between IL-35 and fibrotic diseases for the development of new therapeutic approaches.

## IL-35 and HBV-Related HCC

Similar with the roles of IL-35 in CHB and LC, IL-35 appeared to exhibit immunosuppressive effects in HBV-related HCC. Several studies showed that high IL-35 was associated with poor prognosis in several malignancies, including HCC and gastric cancer (Friedman and Liao, 2015; Fu et al., 2016; Lumley et al., 2018; Teymouri and Pirro, 2018). Up to now, IL-35 played essential roles in restricting anti-cancer immunity and the T cell dysfunction in the tumor microenvironment. Turnis et al. (2016) revealed that Tregs derived IL-35 promoted the expression of several inhibitory receptors such as PD-1, LAG-3, and TIM-3, which then promoted the T cell exhaustion in tumor. In contrast, neutralization with an IL-35-specific antibody or Tregs-restricted deletion of IL-35 production limited tumor growth in multiple murine models of human cancer. Restriction of IL-35 level in tumor tissues contributed to the proliferation of Teffs, as well as their effecting function and the antigenic specificity. Therefore, Tregs and the associated cytokines formed the major barrier for the immunity against tumor.

In HCC tumor tissues, high IL-35 expression was associated with tumor cell invasion and poor prognosis. It was also an independent prognostic factor for HCC recurrence (Long et al., 2016; Damo and Joshi, 2019). To date, little is known about the roles of IL-35 in the pathogenesis of HCC. Secretion of IL-35 by liver tumor cells has been reported to associate with IL-35 up-regulation via a positive feedback, inhibition of proliferation, activation, cytotoxicity of CD8^+^ T cells that promoted disease progression, and viral gene mutation and induction of tumor immune escape (Qiu et al., 2016; Damo and Joshi, 2019; Xue et al., 2019). IL-35 regulated the tumor microenvironment together with negative regulators (e.g., IL-18BP and IL-10), which played a key role in tumor immune escape, and promoted tumor progression and metastasis. It is an important factor in promoting tumorigenesis and development, and reduction of IL-35 may facilitate the control of disease progression (Xiang and Xie, 2015; Long et al., 2016). Meanwhile, it could limit the infiltration, effector function, and immune memory of antigen-specific T cells and promote the expression of various immunosuppressive receptors (e.g., PD-1, TIM3, CTLA-4, and LAG3), which thereby promoted T cell exhaustion in the tumor, and assisted tumor immune escape, tumor proliferation and metastasis (Vignali and Kuchroo, 2012; Fu et al., 2016; Sawant and Yano, 2019).

In tumor tissues, IL-35 involved in JAK/STAT pathway phosphorylation by recruiting bone marrow-derived CD11b^+^Gr1^+^ inhibitory effector cells, which then promoted the tumor angiogenesis and immunosuppression of the tumor microenvironment (Facciabene et al., 2012; Liang et al., 2016; Long et al., 2016; Turnis et al., 2016). Therefore, the immunosuppressive activity and anti-inflammatory properties of IL-35 may be attributed to its potential in inhibiting Teffs amplification, as well as promoting the generation of IL-35 secreted iTr35 and Bregs in the tumor environment. Besides, it promoted host immune tolerance to infection and tumor cells, which played crucial roles in the pathogenesis of various diseases. This indicates that IL-35 may be a new target for the treatment of infectious diseases, malignancies, and inflammatory diseases.

## Possible Immunoregulatory Mechanism of IL-35 in CHB

**FIGURE 4:**
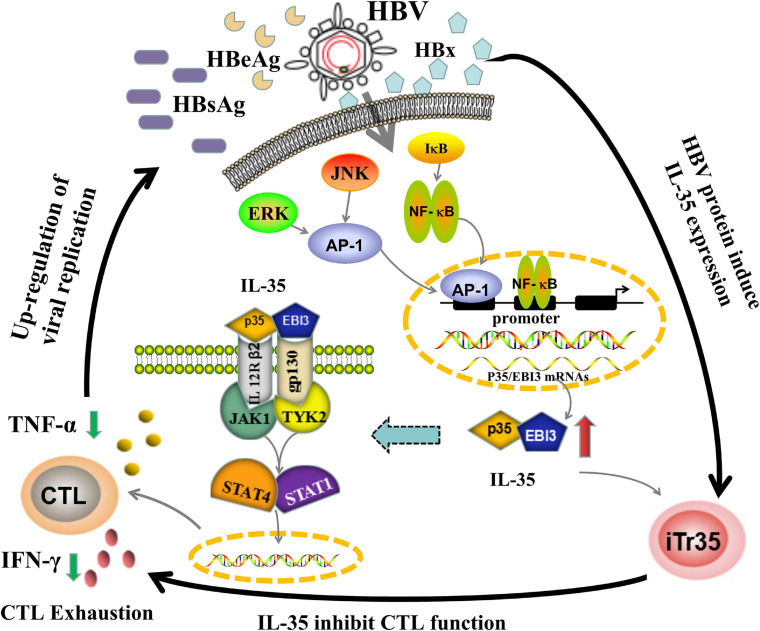
The potential working model of IL-35 immunepathogenesis in chronic HBV infection. The mechanisms of IL-35 mediated immune-suppressive regulation in CHB may be linked to both upstream and downstream pathways. When HBV escaped the host immune system, it would trigger the release of HBV related protein especially HBx, which subsequently activated ERK/AP-1 and IκB/NF-κB signaling pathway to up-regulate IL-35 expression. The increase of IL-35 would bind to IL-12Rβ2/gp130 heterodimers receptors, yielding the formation of a tetramer. After combining to JAK1 and TYK2 kinases, the tetramer was be phosphorylated and eventually activated STAT1/STAT4 pathways by phosphorylation of their transcription factor, which tailed the HBV-specific CTLs into a state of exhaustion. The decrease of number and function of CTLs would further induce the development of chronic HBV infection. The immunological roles of IL-35 were closely related to the etiology of CHB.

As mentioned above, although there are various studies on the role of IL-35 in liver diseases, including CHB, LC, and HCC (Table 2), little is known about how IL-35 affects the HBV replication. To illustrate related mechanism, further researches are needed to investigate the immunopathology of IL-35. Currently, the immunoregulatory function of IL-35 can be depicted based on known upstream and downstream pathways (Figure 4). Downstream of IL-35, EBI3, and p35 subunits had been shown to form a tetramer by binding to the corresponding receptors (i.e., gp130 and IL-12Rβ2). In turn, intracellular JAK kinase was phosphorylated, which ultimately induced the phosphorylation of the downstream STAT transcriptional factor and certain gene expression upon translocation to a specific promoter site gene expression. The specific signaling pathway may be associated with the function of IL-35 (Olson et al., 2013; Egwuagu et al., 2015; Trehanpati and Vyas, 2017). In previously published study, the inhibition of Teffs proliferation and function mediated by IL-35 *in vitro* was in a dose-dependent manner, while activation of the STAT pathway was reported to participate in this process (Collison et al., 2010). However, our recent study suggested that IL-35 could activate the JAK1/TYK2-STAT1/STAT4 pathway in CTLs *in vitro*, while IFN-γ and TNF-α expression showed increase in CTLs when blocking the JAK-STAT pathway. In addition, the expression of the inhibitory receptors (e.g., PD-1, CTLA-4 and LAG-3) on CTLs was down-regulated in the presence of JAK-STAT pathway blockage (Dong et al., 2020). IL-35 triggered the inhibition of HBV-specific CTLs function by modulating the JAK1/TYK2-STAT1/STAT4 pathway. Meanwhile, JAK-STAT pathway block could recover the function of CTL. In this regard, blockage of IL-10, IL-35, and IL-35 signaling pathways (e.g., JAK1 or STAT1) would lead to restore of Teffs function. This indeed contributed to HBV clearance, which may provide a new experimental basis for immunotherapy for CHB.

**TABLE 2:** Immunoregulatory activities of IL-35 in HBV-related liver diseases.

Disease	Action	Role	Outcomes reported	References
CHB	Significantly increased; Positively correlated with HBV DNA load and Tregs levels	Modulate Tregs/Th17 balance; Induction of effector T-cells exhaustion and immunosuppression; Stimulating HBV transcription and replication	Lead to imbalance of immune response and contribute to viral persistence	Li et al., 2015; Zhou et al., 2015; Teng et al., 2019; Yang et al., 2019
HBV-related LC	Significantly increased; Negatively correlated with the albumin level and the Child-Pugh score	Augment and extend hepatic inflammation; Sustaining the pathologic process	Enhance the tolerance to infection, trigger the disease progression	Cheng et al., 2015; Shi et al., 2015; Sun et al., 2012; Slawek et al., 2020
HBV-related HCC	Notably elevated; Positively correlated with Treg infiltration and HCC aggressiveness; Negatively correlated with TNM stage, histological grade, tumor size, vascular invasion and lymph node metastasis	Reducing perforin expression and IFN-γ production, elevating PD-1 and CTLA-4 expression; Trigger the decline of effector T-cells and the functional attenuation	Limit effective anti-tumor immunity	Fu et al., 2016; Long et al., 2016; Qiu et al., 2016; Damo and Joshi, 2019

*CHB, Chronic hepatitis B; LC, Liver cirrhosis; HCC, Hepatocellular carcinoma; TNM, Tumor node metastasis; IFN-γ, Interferon-gamma; PD-1, Programmed cell death protein 1; CTLA-4, Cytotoxic T Lymphocyte-associated antigen-4.*

More studies are needed to elucidate the possible mechanisms involved in HBV-triggering production of IL-35. Study from Liu et al. (2011) provided first evidence that HBV may act as an inducer for the CD4^+^ T cells to initiate IL-35 secretion based on the data that both the mRNA expression of EBI3/p35 and the protein secretion of IL-35 were detectable in circulating CD4^+^ T cells in patients with chronic hepatitis B. However, there remains a lack of research on IL-35 derived from Breg cells in HBV infection. Future investigations are needed to fully elucidate the molecular mechanism of how HBV trigger IL-35 secretion in persistent HBV infection and its related liver deterioration. With further advances in the understanding of IL-35 and its immunosuppressive mechanisms, it will be possible to design targeted immunotherapies and antivirus approaches to modulate the immune response for controlling persistent chronic HBV infection or deterioration in liver diseases.

HBV related proteins (e.g., HBx, HBs, HBc, HBe, preS1, and preS2) may be upstream protein associated with the IL-35 secretion. The *HBx* gene encoding the HBx protein was recognized as one of the most essential determinants for viral replication, dissemination, and viral-medicated pathogenesis (Slagle and Bouchard, 2018). The HBx protein served as a multifunctional HBV regulator that interacted with multiple protein kinases in the cytosol. Secondly, it could act as a kinase activator or co-stimulatory molecule, which then directly activated target cellular promoters and enhancers to increase viral gene expression and replication. Taken together, HBx protein participated in intracellular signal transduction, colonization and transformation, and inhibited the hepatocellular apoptosis, and invasion and metastasis of HCC (Bagga et al., 2016). In the pathogenesis of CHB, HBx could activate ERK and NF-κB signaling pathways, and then triggered the up-regulation of IL-23 and over-expression of the transcription factor AP-1 of ERK/JNK signaling pathway that contributed to the proliferation of hepatoma cells (Shan et al., 2010; Xia et al., 2012). Also, it was implicated with the metabolism of arachidonic acid and activation of the ERK signaling pathway and its positive feedback loop, which subsequently promoted the pathogenesis of hepatic inflammation (Cho et al., 2015). Recently, it has been proposed that there are binding sites for AP-1 and NF-κB transcription factors in the p35/EBI3 subunit gene promoters. In particular, NF-κB can directly activate the *EBI3* gene promoter in bone marrow-derived DCs (Li et al., 2012; Song and Ma, 2016). Therefore, up-regulation of IL-35 expression in CHB patients may be mediated by the HBx/ERK/NF-κB pathway, which inhibited the function of Teffs and attenuated the antiviral immunity in hosts. Considering the roles of IL-35 on the receptors and/or signaling pathways during HBV infection, it is reasonable to speculate that IL-35 may serve as a novel immunotherapy target for chronic HBV infection related diseases.

## Challenges and Prospects

HBV infection has caused great health problems and heavy economic burdens. Oral administration of nucleos(t)ide analogs is the main treatment option for anti-HBV therapy with most cases achieving a high virological suppression. For example, in patients treated with entecavir and tenofovir, more than 95% of patients’ serum HBV DNA were below the detection limit. In addition, the long-term suppression of the virus by nucleo(t)ide analog therapy also resulted in significant improvement in liver histology and reduced the incidence of liver cirrhosis, as well as hepatocellular carcinoma (Wong et al., 2013; Wu et al., 2014; Seto et al., 2018). Its main disadvantages include the necessity of lifelong treatment, difficulty in the clearance of HBsAg in serum and the possibility of HBV reactivation. Therefore, there is an urgent need to explore new anti-inflammatory points and drug targets in order to establish effective treatment options. For the immunoregulatory therapy in treating HBV infection, it is necessary to develop regimens based on blocking checkpoint inhibitor, specific T cell vaccines and genetically engineered T cells such as chimeric antigen receptor (CAR) and TCR redirected T cells. These contribute to the restoration of aberrant HBV specific responses, which promoted the clearance of serum HBsAg (Ye et al., 2015; Boni et al., 2019a; Bertoletti and Tan, 2020). Previous studies suggested that intervention strategies may be helpful to the recovery of depleted T cells by blocking co-inhibitory pathways (Boni et al., 2019a; Meng et al., 2020). However, most of them are in the preclinical or phase I or II stage. These therapies include HBV-specific immunomodulators, such as immunostimulants [e.g., Inarigivir (SB 9200) and the anti-PD-1 antibody nivolumab] and therapeutic vaccines (e.g., GS-4774 and TG-1050) (Martin et al., 2017; Verdon et al., 2017; Yuen et al., 2018; Boni et al., 2019b). A novel CAR construct is developed to exert a profound influence on the expansion, differentiation, development, and survival of lymphocytes (Boni et al., 2019a; Bertoletti and Tan, 2020). Kruse et al. (2018) also reported that HBsAg-CAR T cells have anti-HBV activity in an authentic preclinical HBV infection animal model. In addition, this new immunoregulatory therapy in treating HBV infection can reduce the HBsAg concentration to varying degrees, and induce HBsAg serum clearance in a few cases. Above experimental evidences suggest that immune-based therapies constitute a promising immune modulatory approach for a therapeutic restoration of protective immunity and serve as a new direction of current research in the field of liver diseases. Therefore, due to the IL-35 play a unique role in the IL-12 family and show immunosuppressive activities on Teffs during HBV infection. Assessing the tissue and cellular origins of IL-35 during chronic HBV infection will help determine its potential as a biomarker for the diagnosis and prognostic evaluation of HBV. Elucidation of the immunopathologic roles of IL-35 in the progression of CHB may contribute to promise candidates for the development of new immunotherapeutic strategies targeting IL-35.

## Author Contributions

XLi drafted and revised the manuscript. XLiu participated in data collection and modified the figures. WW was critically revised and given the final approval of the version to be published. All authors have read and approved the article.

## Conflict of Interest

The authors declare that the research was conducted in the absence of any commercial or financial relationships that could be construed as a potential conflict of interest.
